# Supplementary material for: HIVprotI: an integrated web based platform for prediction and design of HIV proteins inhibitors
Source: J Cheminform. 2018 Mar 9;10:12. doi: 10.1186/s13321-018-0266-y (PMC5845081; doi:10.1186/s13321-018-0266-y)
Supplement: Supplementary file 2 — Additional file 2. Source code of HIVProtI web server. [file 13321_2018_266_MOESM2_ESM.zip › HIVprotI_Source-code/hivprotI/weka-3-6-6/changelogs/CHANGELOG-3-5-7.html]

Weka-3-5-7 Changelog


# Weka-3-5-7 Changelog

Designed for use with Apache Ant.

---

2007-12-17 01:29 mhall

```
Can now make use of attribute weights
```

- classifiers/bayes/NaiveBayes.html (1.22)

2007-12-16 22:17 fracpete

```
moved the remaining dynamic discovery of classes (via ClassDiscovery.find) into GenericPropertiesCreator.props/GenericObjectEditor.props. This should enable environments without the possibility of dynamic class discovery, e.g., Eclipse, to use these classes as well (as long GenericObjectEditor.props is maintained)
```

- core/converters/ConverterUtils.html (1.14)
- core/stemmers/SnowballStemmer.html (1.7)
- gui/visualize/PrintableComponent.html (1.8)

2007-12-16 22:17 fracpete

```
moved the remaining dynamic discovery of classes (via ClassDiscovery.find) into GenericPropertiesCreator.props/GenericObjectEditor.props. This should enable environments without the possibility of dynamic class discovery, e.g., Eclipse, to use these classes as well (as long GenericObjectEditor.props is maintained)
```

- core/ClassDiscovery.html (1.6)
- gui/GenericObjectEditor.html (1.58)
- gui/GenericObjectEditor.props (1.118)
- gui/GenericPropertiesCreator.props (1.17)
- gui/experiment/DatasetListPanel.html (1.26)
- gui/experiment/OutputFormatDialog.html (1.8)
- gui/experiment/ResultsPanel.html (1.43)
- gui/explorer/ClassifierPanel.html (1.110)

2007-12-13 21:32 mhall

```
Changed so that only the weka.jar goes into the OS X application as a resource, all other files go into a separate directory in the disk image
```

- build.xml (1.41)

2007-12-13 02:45 mhall

```
branches:  1.1.2;  1.1.4;
Initial import of Mac OS X icon
```

- gui/weka\_icon.icns (1.1)

2007-12-12 21:40 fracpete

```
branches:  1.1.2;
filter for selection a random subset of attributes, either percentage or absolute number of attributes
```

- filters/unsupervised/attribute/RandomSubset.html (1.1)

2007-12-12 21:03 mhall

```
Fixed return value for accessor method for sample size.
```

- filters/unsupervised/instance/ReservoirSample.html (1.2)

2007-12-10 20:22 fracpete

```
max number of rows to display gets only output in the infopanel, if we really hit the max number of rows
```

- gui/sql/SqlViewer.html (1.6)

2007-12-04 00:29 mhall

```
Added an option for using a user-supplied threshold value
```

- classifiers/meta/ThresholdSelector.html (1.40)

2007-12-01 03:57 fracpete

```
initModel is now also called in setModelFile(File) and getCapabilities(), otherwise the capabilities cannot be determined correctly:
https://list.scms.waikato.ac.nz/mailman/htdig/wekalist/2007-December/011783.html
```

- classifiers/misc/SerializedClassifier.html (1.3)

2007-11-28 03:04 mhall

```
Initial import of Martin's feature selection stuff.
```

- attributeSelection/LFSMethods.html (1.1)
- attributeSelection/LinearForwardSelection.html (1.1)
- attributeSelection/SubsetSizeForwardSelection.html (1.1)

2007-11-27 03:30 mhall

```
Added Martin Guetlein's bug fix for merit caching
```

- attributeSelection/BestFirst.html (1.28)

2007-11-23 02:44 mhall

```
Fixed a potential division by zero in out-of-bag routine
```

- classifiers/meta/Bagging.html (1.40)

2007-11-21 22:37 fracpete

```
added capability MISSING_CLASS_VALUES (kernels don't care about class)
```

- classifiers/functions/supportVector/PolyKernel.html (1.7)
- classifiers/functions/supportVector/Puk.html (1.3)
- classifiers/functions/supportVector/RBFKernel.html (1.9)
- classifiers/functions/supportVector/StringKernel.html (1.7)

2007-10-30 03:27 mhall

```
When saving trained model to binary file now saves training header (like the Explorer does). Similarly, when loading binary model attempts to load the header from the stream as well.
```

- classifiers/Evaluation.html (1.83)

2007-10-29 20:34 fracpete

```
support of an attribute range to base the distance calculation on is now mandatory for all distance functions
```

- core/DistanceFunction.html (1.7)

2007-10-28 23:41 fracpete

```
new distance functions (Manhattan and Chebyshev) which share the same abstract superclass as the Euclidean distance function: NormalizableDistance
```

- core/EuclideanDistance.html (1.12)

2007-10-28 23:41 fracpete

```
branches:  1.1.2;
new distance functions (Manhattan and Chebyshev) which share the same abstract superclass as the Euclidean distance function: NormalizableDistance
```

- core/ChebyshevDistance.html (1.1)
- core/ManhattanDistance.html (1.1)
- core/NormalizableDistance.html (1.1)

2007-10-28 23:31 fracpete

```
added scale and transformation parameter to get different range output than [0,1]
```

- filters/unsupervised/attribute/Normalize.html (1.12)

2007-10-25 01:59 fracpete

```
branches:  1.3.2;
PCA filter based on the attribute selection evaluator weka.attributeSelection.PrincipalComponents
```

- filters/unsupervised/attribute/PrincipalComponents.html (1.3)

2007-10-09 23:08 mhall

```
Now throws an exception if NaN occurs during standardization. This avoids introducing missing values into the filtered data set in this situation.
```

- filters/unsupervised/attribute/Standardize.html (1.11)

2007-10-06 22:44 fracpete

```
removed "final" from getOutputFormat method declaration since that caused VerifyError exceptions in derived filters on Mac Books:
https://list.scms.waikato.ac.nz/mailman/htdig/wekalist/2007-October/011339.html
```

- filters/unsupervised/attribute/PotentialClassIgnorer.html (1.7)

2007-09-20 21:43 fracpete

```
test for missing values missed the last column (in case it wasn't the class attribute!)
```

- core/Capabilities.html (1.24)

2007-09-10 21:53 fracpete

```
mathematical expression got only evaluated for toString/getMeasure, but for actual setup of filter/classifier. Didn't pop up as error since most Weka schemes do NOT check the input in the get-method (you only get wonky results). Was discovered by M. Fatih Akay using LibSVM, since LibSVM threw an exception in case of invalid parameters, but the values would have been valid IF the expression would have been evaluated.
```

- classifiers/meta/GridSearch.html (1.8)

2007-09-07 07:05 fracpete

```
classifyInstance returned the probability instead of the index from the class probability array for nominal classes (in case of average/product/majority/min/max rule). Thanks to Samuel Robert Reid
```

- classifiers/meta/Vote.html (1.18)

2007-09-07 07:02 fracpete

```
tokenizer could not be serialized due to unserializable StringTokenizer class. changed m_Tokenizer to transient.
```

- core/tokenizers/WordTokenizer.html (1.2)

2007-09-03 22:11 fracpete

```
removed classifyInstance method, since there were discrepancies between the labels returned by classifyInstance and the best one returned by distributionForInstance (svm_predict returned other labels than svm_predict_probability???)
used version of libsvm: 2.84
```

- classifiers/functions/LibSVM.html (1.12)

2007-08-30 23:11 mhall

```
Fixed bug that prevented quoted ? from being parsed as a label rather than a missing value
```

- core/converters/CSVLoader.html (1.16)

2007-08-26 23:08 mhall

```
Fixed inconsistent naming of toolTipText method
```

- classifiers/bayes/AODEsr.html (1.2)

2007-08-23 02:25 mhall

```
Fixed bug that caused stack overflow by removing all uses of Utils.sm and Utils.gr
```

- classifiers/trees/RandomTree.html (1.19)

2007-08-22 02:00 mhall

```
Initial import. Contributed by Geoff Webb's group.
```

- classifiers/trees/J48graft.html (1.1)
- classifiers/trees/j48/C45PruneableClassifierTreeG.html (1.1)
- classifiers/trees/j48/GraftSplit.html (1.1)

2007-08-20 00:58 fracpete

```
stopwords no longer get ignored
```

- filters/unsupervised/attribute/StringToWordVector.html (1.20)

2007-08-09 22:36 fracpete

```
savers were too restrictive in writeBatch method: instead ALWAYS having a file to write to, one needs only either a file OR a stream (both are used through a Writer)
```

- core/converters/ArffSaver.html (1.7)
- core/converters/CSVSaver.html (1.8)
- core/converters/LibSVMSaver.html (1.3)
- core/converters/XRFFSaver.html (1.4)

2007-08-01 04:06 fracpete

```
added general Jython support
Jython classes that are derived from weka.classifiers.Classifier can be executed with the wrapper classifier weka.classifiers.JythonClassifier (serializing/deserializing a trained model is unfortunately NOT possible, a call of buildClassifier is always necessary after deserialization)
```

- core/SerializedObject.html (1.11)

2007-08-01 04:06 fracpete

```
branches:  1.1.2;
added general Jython support
Jython classes that are derived from weka.classifiers.Classifier can be executed with the wrapper classifier weka.classifiers.JythonClassifier (serializing/deserializing a trained model is unfortunately NOT possible, a call of buildClassifier is always necessary after deserialization)
```

- classifiers/JythonClassifier.html (1.1)
- core/Jython.html (1.1)
- core/JythonObject.html (1.1)
- core/JythonSerializableObject.html (1.1)

2007-07-15 21:36 mhall

```
Instance variable holding an Evaluation object was not set to null after buildClassifier, resulting in not serializable exception in Experimenter
```

- classifiers/rules/DecisionTable.html (1.44)

2007-07-11 21:52 fracpete

```
added the "output of additional attributes" option to the "more options" dialog in order to output attribute values alongside the predictions (just like on the commandline with "-p")
```

- gui/explorer/Explorer.props (1.6)
- gui/explorer/ExplorerDefaults.html (1.4)

2007-07-09 02:41 cvs\_rrb

```
Fix related to Markov blanket correction
```

- classifiers/bayes/net/search/global/K2.html (1.7)
- classifiers/bayes/net/search/global/SimulatedAnnealing.html (1.5)

2007-07-09 02:41 cvs\_rrb

```
Fix related to Markov blanket correction
```

- classifiers/bayes/net/search/local/K2.html (1.7)
- classifiers/bayes/net/search/local/SimulatedAnnealing.html (1.5)

2007-07-09 02:38 cvs\_rrb

```
Markov blanket correction fix
```

- classifiers/bayes/net/search/SearchAlgorithm.html (1.8)

2007-07-03 23:48 mhall

```
Instance weights were being cast to ints for some reason. Removed the casts
```

- classifiers/bayes/AODE.html (1.18)

2007-07-03 01:24 mhall

```
Fixed bug in kthSmallestValue that prevented the largest value from being returned
```

- core/Instances.html (1.73)

2007-07-02 04:10 cvs\_rrb

```
Access to nodes (usefull for Bayes network GUI)
```

- gui/graphvisualizer/HierarchicalBCEngine.html (1.6)
- gui/graphvisualizer/LayoutEngine.html (1.6)

2007-07-02 03:59 cvs\_rrb

```
Initialize from Editable Bayes network
```

- classifiers/bayes/net/BayesNetGenerator.html (1.13)

2007-07-02 03:58 cvs\_rrb

```
Access from Bayes network GUI
```

- classifiers/bayes/net/BIFReader.html (1.13)

2007-07-02 03:58 cvs\_rrb

```
Bayes network algorithm for calculating marginal distributions
```

- classifiers/bayes/net/MarginCalculator.html (1.1)

2007-07-02 03:54 cvs\_rrb

```
Icons for Bayes netowrk GUI
```

- classifiers/bayes/net/icons/alignbottom.png (1.1)
- classifiers/bayes/net/icons/alignleft.png (1.1)
- classifiers/bayes/net/icons/alignright.png (1.1)
- classifiers/bayes/net/icons/aligntop.png (1.1)
- classifiers/bayes/net/icons/arrow-s.png (1.1)
- classifiers/bayes/net/icons/arrow.png (1.1)
- classifiers/bayes/net/icons/centerhorizontal.png (1.1)

2007-07-02 03:54 cvs\_rrb

```
Icons for Bayes netowrk GUI
```

- classifiers/bayes/net/icons/centervertical.png (1.1)
- classifiers/bayes/net/icons/copy.png (1.1)
- classifiers/bayes/net/icons/cut.png (1.1)
- classifiers/bayes/net/icons/help.png (1.1)
- classifiers/bayes/net/icons/layout.png (1.1)
- classifiers/bayes/net/icons/line-s.png (1.1)
- classifiers/bayes/net/icons/line.png (1.1)
- classifiers/bayes/net/icons/lines-s.png (1.1)
- classifiers/bayes/net/icons/lines.png (1.1)
- classifiers/bayes/net/icons/new.png (1.1)
- classifiers/bayes/net/icons/open.png (1.1)
- classifiers/bayes/net/icons/oval-s.png (1.1)
- classifiers/bayes/net/icons/oval.png (1.1)
- classifiers/bayes/net/icons/paste.png (1.1)
- classifiers/bayes/net/icons/print.png (1.1)
- classifiers/bayes/net/icons/rect-s.png (1.1)
- classifiers/bayes/net/icons/rect.png (1.1)
- classifiers/bayes/net/icons/redo.png (1.1)
- classifiers/bayes/net/icons/rrect.png (1.1)
- classifiers/bayes/net/icons/save.png (1.1)
- classifiers/bayes/net/icons/select-s.png (1.1)
- classifiers/bayes/net/icons/select.png (1.1)
- classifiers/bayes/net/icons/spacehorizontal.png (1.1)
- classifiers/bayes/net/icons/spacevertical.png (1.1)
- classifiers/bayes/net/icons/undo.png (1.1)
- classifiers/bayes/net/icons/zoomin.png (1.1)
- classifiers/bayes/net/icons/zoomout.png (1.1)

2007-07-02 03:51 cvs\_rrb

```
Bayes network GUI
```

- classifiers/bayes/net/EditableBayesNet.html (1.1)
- classifiers/bayes/net/GUI.html (1.1)

2007-07-02 03:49 cvs\_rrb

```
access for Bayes net GUI
```

- classifiers/bayes/net/ParentSet.html (1.7)

2007-07-02 03:45 cvs\_rrb

```
Access for Bayes Net GUI
```

- gui/graphvisualizer/GraphNode.html (1.5)

2007-07-02 03:44 cvs\_rrb

```
Access for EditableBayesNet
```

- classifiers/bayes/BayesNet.html (1.31)

2007-07-01 22:42 mhall

```
Sebastian has given copyright to UoW
```

- core/tokenizers/NGramTokenizer.html (1.2)

2007-06-29 04:00 fracpete

```
disabled EnsembleLibrary for now due to bugs: craps out if the GOE-editable-object depth is more than 2, e.g., a FilteredClassifier with SMO (the kernel is the third level) generates a StackOverFlowError
```

- gui/GUIChooser.html (1.33)

2007-06-29 03:42 fracpete

```
disabled EnsembleLibrary for now due to bugs: craps out if the GOE-editable-object depth is more than 2, e.g., a FilteredClassifier with SMO (the kernel is the third level) generates a StackOverFlowError
```

- gui/Main.html (1.13)

2007-06-26 21:05 fracpete

```
fixed technical information (booktitle -> journal)
```

- classifiers/trees/LMT.html (1.11)

2007-06-26 04:19 mhall

```
branches:  1.1.2;
file movePublicDomain.sh was initially added on branch waikato.
```

- scripts/movePublicDomain.sh (1.1)

2007-06-25 22:50 mhall

```
This class has been placed into the public domain by the author, Werner Randelshofer
```

- gui/SplashWindow.html (1.3)

2007-06-25 02:05 fracpete

```
de-serializing savers no longer sets the file, since the KnowledgeFlow handles that itself, by using prefix, directory and relation-name for the filename
```

- gui/beans/xml/XMLBeans.html (1.8)

2007-06-25 02:02 fracpete

```
statement variables are now transient (otherwise an exception is raised that these objects aren't serializable)
added checks whether a DB connection is active (after de-serializing for instance)
```

- experiment/DatabaseUtils.html (1.32)

2007-06-25 02:01 fracpete

```
added checks whether a DB connection is active (after de-serializing for instance)
```

- core/converters/DatabaseConnection.html (1.7)

2007-06-25 01:59 fracpete

```
doesn't stumble over '' anymore by replacing it with one single quote (creates an invalid SQL statement)
```

- core/converters/DatabaseSaver.html (1.11)

2007-06-25 01:57 fracpete

```
removed restriction that nominal attributes have to have at least one attribute. ARFF files generated from databases can have empty nominal attributes, e.g., "Summary" from a results table of the Experimenter may not be filled in (only missing values). Such a table could not be loaded again in the Explorer again.
```

- core/converters/ArffLoader.html (1.19)

2007-06-22 04:35 mhall

```
UoW has the copyright.
```

- classifiers/functions/LeastMedSq.html (1.16)

2007-06-22 01:25 fracpete

```
added information about how ROC and AUC are computed, based on this Wekalist post:
https://list.scms.waikato.ac.nz/mailman/htdig/wekalist/2007-June/010386.html
```

- classifiers/evaluation/ThresholdCurve.html (1.22)

2007-06-21 23:24 mhall

```
Changed copyright to University of Waikato as Len gave over the copyright.
```

- classifiers/lazy/kstar/KStarCache.html (1.10)
- classifiers/lazy/kstar/KStarConstants.html (1.7)
- classifiers/lazy/kstar/KStarNominalAttribute.html (1.6)
- classifiers/lazy/kstar/KStarNumericAttribute.html (1.6)
- classifiers/lazy/kstar/KStarWrapper.html (1.6)

2007-06-21 23:13 mhall

```
Changed copyright notice as Alex has given copyright to the University
```

- classifiers/meta/Grading.html (1.12)
- classifiers/meta/StackingC.html (1.14)

2007-06-21 22:54 mhall

```
branches:  1.3.2;
Changed copyright back to Haijian as he was not on a scholarship when this code was written.
```

- classifiers/trees/BFTree.html (1.3)
- classifiers/trees/SimpleCart.html (1.3)

2007-06-21 22:17 fracpete

```
added command "history" to list the current conent of the command history
last X number of commands are saved in $HOME/SimpleCLI.props (X = HistorySize in props file)
```

- gui/SimpleCLI.props (1.1)
- gui/SimpleCLIPanel.html (1.5)

2007-06-20 23:02 fracpete

```
instead of listing only number of test instances, the split evaluators now list number of training and test instances (like the ClassifierSplitEvaluator)
```

- experiment/CostSensitiveClassifierSplitEvaluator.html (1.17)
- experiment/RegressionSplitEvaluator.html (1.24)

2007-06-20 04:12 fracpete

```
one Utils.quote call too much
```

- filters/Filter.html (1.41)

2007-06-20 01:39 fracpete

```
source code generation now generates a static Weka filter
```

- filters/AllFilter.html (1.12)
- filters/CheckSource.html (1.2)
- filters/unsupervised/attribute/Center.html (1.4)
- filters/unsupervised/attribute/ReplaceMissingValues.html (1.10)

2007-06-19 04:28 fracpete

```
fixed "sourcing" of classifiers:
- Explorer outputs now the complete class, not just the static class, but also the wrapper class
- wrapper class has now a main method and a toString method to enable calls from the commandline like with any other classifier
- reversed all "public class" to "class" again
- class attribute now gets set to missing explicitly before the static class is called ("classify")
- updated weka.classifiers.CheckSource to reflect changes (source code has to be derived from weka.classifiers.Classifier now)
- fixed OneR's toSource method
```

- classifiers/CheckSource.html (1.3)
- classifiers/meta/AdaBoostM1.html (1.39)
- classifiers/meta/LogitBoost.html (1.42)
- classifiers/rules/OneR.html (1.25)
- classifiers/rules/ZeroR.html (1.18)
- classifiers/trees/DecisionStump.html (1.25)
- classifiers/trees/Id3.html (1.22)
- classifiers/trees/J48.html (1.8)

2007-06-19 00:52 fracpete

```
added new interface weka.filters.Sourcable and let a few simple filters implement it to generate source code (option "-z classname")
```

- filters/Sourcable.html (1.1)

2007-06-19 00:46 fracpete

```
fixed typo in Javadoc
```

- classifiers/Sourcable.html (1.6)

2007-06-18 21:07 fracpete

```
fixed toString() method if no model built yet
```

- classifiers/mi/CitationKNN.html (1.7)

2007-06-18 01:54 fracpete

```
fixed tilde in BibTeX Javadoc output
```

- classifiers/trees/UserClassifier.html (1.28)
- core/neighboursearch/kdtrees/SlidingMidPointOfWidestSide.html (1.2)
- datagenerators/classifiers/classification/LED24.html (1.5)
- experiment/PairedCorrectedTTester.html (1.12)
- filters/unsupervised/attribute/RELAGGS.html (1.2)

2007-06-18 01:54 fracpete

```
branches:  1.3.2;
fixed tilde in BibTeX Javadoc output
```

- classifiers/functions/supportVector/RegSMOImproved.html (1.3)

2007-06-18 01:54 fracpete

```
fixed tilde in BibTeX Javadoc output
```

- classifiers/BVDecompose.html (1.14)
- classifiers/meta/AdditiveRegression.html (1.24)
- classifiers/rules/PART.html (1.9)
- classifiers/rules/Ridor.html (1.20)

2007-06-18 01:54 fracpete

```
branches:  1.65.2;
fixed tilde in BibTeX Javadoc output
```

- classifiers/functions/SMO.html (1.65)

2007-06-18 01:54 fracpete

```
branches:  1.4.2;
fixed tilde in BibTeX Javadoc output
```

- classifiers/functions/SVMreg.html (1.4)

2007-06-18 01:54 fracpete

```
branches:  1.3.2;
fixed tilde in BibTeX Javadoc output
```

- core/neighboursearch/CoverTree.html (1.3)

2007-06-18 01:54 fracpete

```
branches:  1.6.2;
fixed tilde in BibTeX Javadoc output
```

- classifiers/BVDecomposeSegCVSub.html (1.6)

2007-06-18 01:53 fracpete

```
fixed preamble comment indentation
```

- clusterers/Cobweb.html (1.24)

2007-06-18 01:51 fracpete

```
branches:  1.5.2;
fixed Javadoc of -C <cache size> option
```

- classifiers/mi/MISMO.html (1.5)
- classifiers/mi/MISVM.html (1.5)

2007-06-18 01:49 fracpete

```
branches:  1.6.2;
updated Javadoc of -C <cache size> option of SMO
```

- classifiers/meta/Dagging.html (1.6)

2007-06-18 01:47 fracpete

```
branches:  1.13.2;
fixed Javadoc of -C <cache size> option
```

- classifiers/functions/SMOreg.html (1.13)

2007-06-18 01:44 fracpete

```
reversed the escaping of the underscore (only tilde now)
```

- core/TechnicalInformation.html (1.9)

2007-06-17 03:54 fracpete

```
added additional output to the log with setup for the AttributeSelectedClassifier
```

- gui/explorer/AttributeSelectionPanel.html (1.49)

2007-06-15 06:40 fracpete

```
added three new measures to the Experimenter:
  Serialized_Model_Size - size of the serialized classifier object
  Serialized_Train_Set_Size - size of the serialized train set
  Serialized_Test_Set_Size - size of the serialized test set
```

- experiment/ClassifierSplitEvaluator.html (1.28)

2007-06-14 02:09 fracpete

```
fixed serialVersionUID
```

- classifiers/meta/ClassificationViaClustering.html (1.2)

2007-06-14 01:51 fracpete

```
method "mapClasses" is now public and static instead of private
```

- clusterers/ClusterEvaluation.html (1.41)

2007-06-08 01:35 fracpete

```
incorporated changes between Jama 1.0.1 and 1.0.2 in Weka's classes
see also Jama's changelog: http://math.nist.gov/javanumerics/jama/ChangeLog
```

- core/matrix/CholeskyDecomposition.html (1.4)
- core/matrix/SingularValueDecomposition.html (1.4)

2007-06-06 04:24 fracpete

```
minimum number of instances of capabilities is now set to number of bins
```

- classifiers/meta/RegressionByDiscretization.html (1.36)

2007-06-05 21:06 fracpete

```
on Macs the updateCurrentConverter() method crashes with a NullPointerException if the setAcceptAllFileFilterUsed(boolean) method is used before the dialog is even displayed (e.g., in the showSaveDialog(Component) method). Looks like Java 1.5 fires property changes differently on Macs (Win32 and Linux works fine). Thanks to Craig Bruce.
```

- gui/ConverterFileChooser.html (1.8)

2007-06-01 04:32 fracpete

```
updated version
```

- core/version.txt (1.5)
